# Increased risk of periodontal disease and poor oral-related quality of life in fibromyalgia

**DOI:** 10.2340/aos.v85.46621

**Published:** 2026-07-31

**Authors:** Katharine Hopkins, Camilla Ahlgren, Åsa Wahlin, Annarita Antelmi, Magnus Bruze, Cecilia Svedman

**Affiliations:** a Department of Occupational and Environmental Dermatology, Lund University, Skåne University Hospital, Malmö, Sweden; b Department of Research, Development and Education, Public Dental Service, Lund, Sweden; c Department of Periodontology, Public Dental Service, Lund, Sweden

**Keywords:** contact allergy, fibromyalgia, OHIP-14, oral symptoms, periodontitis

## Abstract

**Objective:**

(1) To survey oral health in fibromyalgia (FM) and compare with population controls and (2) to assess whether patterns of contact allergy are related to oral status in FM.

**Materials and methods:**

Participants: 60 females with FM who had previously received contact allergy testing. Controls: 120 age- and gender-matched individuals. Questionnaire on aspects of oral health and symptoms and Oral Health Impact Profile-14. Clinical and radiological examination regarding oral health status.

**Results:**

The degree of periodontal disease differed significantly between the FM and control groups (*p* = 0.017), with the difference most marked in younger patients. No difference was seen regarding dental caries and filled surfaces. FM patients had a higher prevalence of oral lesions than control patients (30.0% vs. 10.1%, *p* < 0.001). Younger FM patients had a poorer oral health-related quality of life than older patients. There was no relationship between oral symptoms or oral lesions and contact allergy.

**Conclusions:**

Patients with FM have a poorer oral status than controls, particularly concerning periodontitis and oral lesions. Further work is required to assess whether contact allergy may play a role in poorer oral status and systemic inflammation in FM.

## Introduction

Fibromyalgia (FM) is a condition characterized by widespread pain with a pervasive high symptom severity [1]. A considerably higher prevalence is reported in women [1, 2]. The modified American College of Rheumatology diagnostic criteria (2016) take into account the presence of multiple somatic symptoms including fatigue, cognitive difficulties, and headaches [1]. In this context, oral symptoms are commonly described in FM, including xerostomia, glossodynia, and temporal joint dysfunction, with the prevalence of oral symptoms being as high as 70% [3]. It has also been noted that medications commonly employed in FM, including antidepressants and analgesics, can cause oral symptoms including dryness and bruxism [4]. Despite the high reported burden of oral symptoms in FM, few studies have reported on oral status in individuals with the disorder. One study reported low prevalence of identified oral lesions in FM, which did not differ from controls, despite a high symptom burden in the FM group [3].

We have reported our studies on contact allergy in the FM population, which demonstrated that females with FM have a higher burden of contact allergy when compared with controls, and that the pattern of contact allergy in this group appears to favor contact allergens where sensitization potentially can occur via the oral mucosa [5–8]. These allergens, including gold and certain flavor/fragrance substances, have also been implicated in systemic contact dermatitis, whereby systemic exposure to an allergen in a sensitized individual can give rise to cutaneous and extracutaneous symptoms such as pain in muscles and joints [9]. In the case of gold, this can be attributed to a low chronic release of gold from dental restorations [10, 11]. Manifestations of gold allergy in gold-allergic individuals upon systemic exposure to gold can include general malaise and pain [12, 13].

The question that arises from these studies is whether individuals with FM have a characteristic oral status that can cause a propensity to sensitization to these substances or if they have a higher burden of allergen exposure, either from oral care habits or presence of dental materials. A broader question is whether systemic contact allergy could be a contributory factor in the symptomology of FM. The aims of the current study were as follows: (1) To survey oral health in FM and compare with population controls and (2) to assess whether patterns of contact allergy are related to oral status in FM.

## Materials and methods

This cross-sectional comparative study was performed in 2022–2023 by the Department of Occupational and Environmental Dermatology (DOED), Malmö, Sweden. Clinical examinations were performed in public dental clinics in the regions of Skåne and Halland in southern Sweden. Ethical approval was obtained from the Swedish Ethical Review Authority (*Etikprövningsmyndigheten*) (2022-01573-01).

### Recruitment

#### Study group: FM

In 2022, all individuals (119 females) who participated in the earlier studies performed by the DOED surveying contact allergy in the FM population were contacted in writing and via telephone and offered participation in the study. Recruitment to the contact allergy study has previously been described in detail [6, 7]. In summary, individuals were recruited from branches of the Swedish FM patient organization (*Fibromyalgiförbundet*) in southern Sweden. As recruited individuals were predominantly female (119/120 recruited individuals), subsequent analyses were performed solely on the female participants. Potential participants in this study received detailed written information on the aim of the study, and that participation would include a questionnaire on general and dental health and oral health- related quality of life, an oral examination performed by a dentist, and a panoramic radiograph.

#### General population controls

Age- and sex-matched controls were drawn from a cross-sectional dental epidemiological study performed in the Swedish city of Jönköping in 2013–2014 [14, 15]. This study was part of a larger project initiated in 1973 to provide a representative picture of the dental health in the population [16]. In 2013, 130 randomly selected individuals in banded age groups from 3 to 80 years were invited to participate in the Jönköping study via letter and telephone where possible. All invitees were informed of the purpose of the study, and that participation would include an oral clinical and radiographic (in adults) examination and a questionnaire about oral health-related quality of life and oral health.

### Questionnaire

#### Study group: FM

All participants filled in a questionnaire regarding aspects of oral health; oral hygiene habits, including dentifrices and mouthwash; experience of previous and current dental care; dental materials present; oral symptoms; triggers for oral symptoms, including avoidance behaviors, if present; and exposure and tolerance to different flavors and fragrances (Supplement 1). The questions regarding oral health and practices were taken from the questionnaire used in the longitudinal study from which the control material was drawn [14]. Questions concerning exposure and sensitivity to potential contact allergens were previously used in questionnaire studies assessing contact allergy in the FM population [8].

The Oral Health Impact Profile (OHIP-14) questionnaire was also completed by participants. The OHIP-14 is a self-completed questionnaire consisting of 14 items subdivided into 7 domains. It measures social impacts of oral problems as a total score index. Questions are answered on a Likert scale: 0 (never), 1 (hardly ever), 2 (occasionally), 3 (fairly often), and 4 (very often). The maximum score is 56, with lower scores indicating better oral health-related quality of life (Supplement 2) [17, 18].

#### General population controls

Participants answered a web-based questionnaire, including questions on oral care habits, dental visits, oral hygiene, and dental habits [14].

### Contact allergy testing

All individuals with FM participating in the study had previously received contact allergy testing as part of a survey on prevalence of contact allergy within the FM population. The method and results of this survey have been described comprehensively in previous manuscripts [6, 7]. All participants received patch testing with the Swedish baseline series and an extended dental series between 2017 and 2018. The results from this testing were used for analysis in this study. Repeated patch testing was not performed as the logistical difficulties of repeating the extensive investigation were judged to outweigh the small likelihood of identifying any new contact allergies.

### Clinical examination

**Figure 1:**
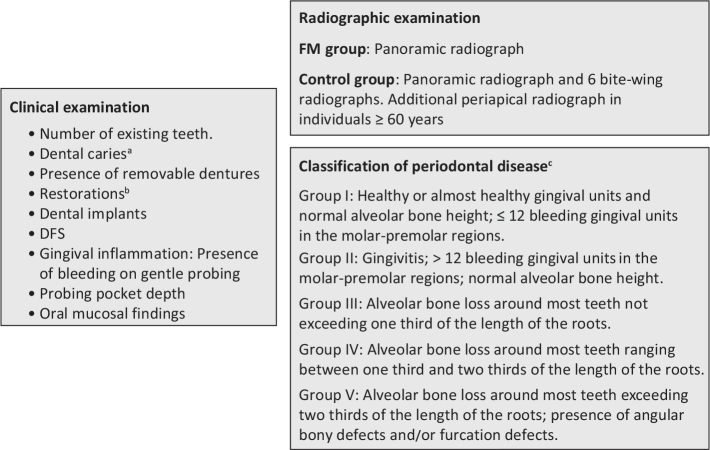
Summary of clinical and radiological examinations performed in the fibromyalgia and control groups in the study. DFS, decayed and filled surfaces [19]; FM, fibromyalgia. ^a^According to the criteria of Koch [20]. ^b^For each tooth surface, presence of amalgam, glass ionomer cement, composite material, gold inlays, metal, or porcelain crowns and bridges. ^c^Classification of periodontal disease using Hugoson and Jordan’s criteria [21].

Details of the clinical and radiological examinations performed are described in Figure 1.

#### Study group: FM

The examinations were performed by two dentists who were both present at all examinations to ensure inter-examiner agreement on examination findings. The dentists were blinded as to whether the participants had a known contact allergy to dental material or taste substances. An oral examination including dental caries, periodontal, and oral mucosal status, as well as registration of dental materials used for rehabilitation, was performed. The clinical examination was supplemented by a panoramic radiograph.

Based on the clinical and radiographic findings, all dentate individuals were classified according to the criteria outlined by Hugoson and Jordan (H&J) [21] (Figure 1).

If any pathology was found on examination, the participants were informed verbally and in writing and advised to contact their regular dentist for treatment.

#### General population controls

Examination was performed between 2013 and 2014 by 11 dentists who underwent information meetings as to the examination and diagnostic criteria.

### Statistical analysis

To enable statistical analysis, the FM participants were grouped into 10-year age categories from 30 to 80 years to match the age categories available from the control group.

Statistical analyses were performed using IBM SPSS Statistics (IBM, Armonk, NY, USA) version 31. Demographic, questionnaire, and clinical data were compared between the FM group and the population controls. Missing data points are excluded from the analyses. Categorical data are presented as number and percentages and age as mean and standard deviation. Statistical analysis was performed using the chi-square or Fisher’s test for categorical data, with post-hoc analysis using the Bonferroni correction. Mann–Whitney U test was used for comparison of means. For analysis of periodontitis, the degree of periodontal disease was regrouped into mild (H&J I–II), moderate (H&J III), and severe (H&J IV–V), and ages were grouped into < 50 years and ≥ 50 years. Negative binomial regression was used to adjust for age in the analysis of caries data. Comparison of OHIP-14 scores between age groups was performed with the Kruskal–Wallis test for nonparametric data. A general linear model was used to assess the effects of grade of periodontal disease and decayed and filled surfaces (DFS) on OHIP scores. *P-*values less than 0.05 were considered statistically significant.

## Results

After the recruitment process, 60 females with FM participated in the study. The FM participants did not differ from the nonparticipants in terms of average age, smoking status, reported oral symptoms, or reported symptoms upon exposure to fragrances or gold jewelry (data available upon request). From the control population, an age-matched sample of 120 females was selected to achieve a ratio of 2:1 between control and FM study patients. The controls were selected via a random number generator within each 10-year age group. Characteristics of the FM and control groups are shown in Table 1.

**Table 1:** Summary of the characteristics of the FM and control groups.

Demographics	Fibromyalgia (%)	Control (%)	*p*
Age, years			
30–39	2	4	
40–49	7	14	
50–59	11	22	
60–69	21	42	
70–79	18	36	
≥ 80	1	2	
Total	60	120	
Mean	58.17 (SD 11.42)	58.17 (SD 11.37)	
Snus (smokeless tobacco)	4 (6.7)	7 (6.0)	1.0
Smoking (current)	4 (6.7)	11 (9.4)	0.78
Education			
Secondary school	14 (23.3)	30 (26.5)	
Higher vocational education	26 (38.2)	42 (37.2)	
University	20 (33.3)	41 (36.3)	
Mean number of teeth	24.65 (SD 3.93)	24.51 (SD 4.87)	0.85

FM: fibromyalgia.

### Oral care habits

All participants in both groups responded that they always use toothpaste when brushing teeth. Regular (daily) use of dental floss/tape was more common in the FM group (73.3% vs. 28.8%, *p* < 0.001), as was regular (daily) use of interdental brushes (65.0% vs. 39.0%, *p =* 0.004). Differences between the groups were also seen when stratified for age, with 70% of FM individuals <50 years using dental floss/tape compared with 29.4% of controls (*p* = 0.09) (individuals ≥ 50 years 74% vs. 28.7%, *p* < 0.001).

### Periodontal disease

Analysis is shown in Table 2. The degree of periodontal disease differed significantly between the FM and control groups (*p* = 0.017). Post-hoc analyses were based on adjusted standardized residuals. A Bonferroni correction was applied for the six cells in the contingency table, resulting in a corrected significance level of α = 0.0083. Mild periodontal disease was significantly more prevalent among controls (47.9%) than among the FM individuals (29.8%), and moderate periodontitis was significantly more common in FM (57.9% vs. 35.3%). Upon analysis solely of the <50-year age group, FM was significantly associated with a higher degree of periodontal disease severity (*p* < 0.001).

**Table 2:** Comparison of periodontal disease severity according to the criteria of Hugoson and Jordan [21] by age (< 50 and ≥ 50 years) in the fibromyalgia and control groups.

Periodontal disease		Fibromyalgia *n* (%)			Controls *n* (%)			*p*
		Total	< 50 years	≥ 50 years	Total	< 50 years	≥ 50 years	
Periodontal disease severity	Mild (H&J I–II)	17 (29.8)^b^	3 (33.0)	14 (29.0)	57 (47.9)^b^	18 (100.0)	39 (38.6)	
	Moderate (H&J III)	33 (57.9)^c^	6 (67.0)	27 (56.3)	42 (35.3)^c^	0	42 (41.6)	
	Severe (H&J IV–V)	7 (12.3)	0	7 (14.6)	20 (16.8)	0	20 (19.8)	
								**0.017** ^**a**^
Total		57	9	48	119	18	101	

n, number; H&J, Hugoson and Jordan.

a Overall Fisher’s exact test.

b,c Significant difference based on analysis of adjusted standardized residuals with Bonferroni correction.

### Oral lesions

The presence of oral mucosal lesions was significantly increased in the FM group (18 (30.0%) vs. 12 (10.0%), *p* < 0.001). Lichenoid lesions were more common in the FM group (7 (11.7%) vs. 5 (4.3%), but this was not significant (*p* = 0.057).

### Dental caries

The number of tooth surfaces with dental caries did not differ between the FM and control groups (2.00 vs. 2.08; *p* = 0.91). There was no significant difference in the percentage of decayed surfaces among remaining teeth between the groups (FM 1.66 (SD 2.59), controls 1.77 (SD 2.18); *p* = 0.91). The mean DFS was 38.47 (SD 21.03) in the FM group and 37.73 (SD 21.57) in the control group (*p* = 0.81). Upon adjustment for age, there was no significant difference between the FM and control groups (incidence rate ratio = 1.03, 95% CI 0.75–1.41, *p* = 0.88).

### OHIP-14

**Figure 2:**
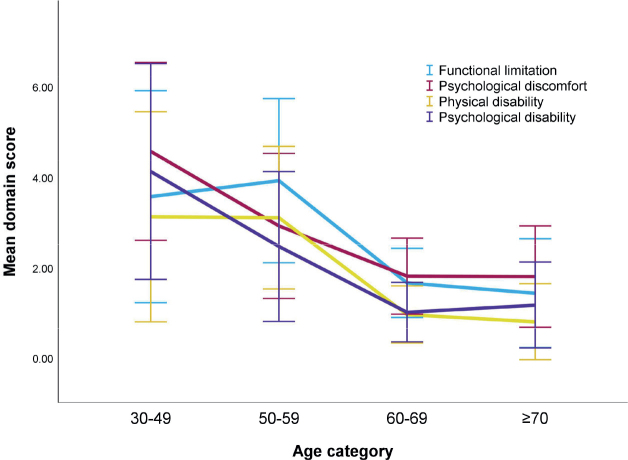
Mean scores of selected domains of the Oral Health Impact Profile-14 questionnaire in the fibromyalgia individuals according to the age group. Maximum score for each domain: eight points. Higher scores represent poorer oral health-related quality of life. Bars represent 95% confidence interval.

For analysis, the FM individuals were reassigned into four groups: 30–49 years, 50–59 years, 60–69 years, and ≥ 70 years. The mean OHIP-14 score in the FM group was 14.61 (SD 13.85). The mean OHIP-14 score in the 30–49 years group was 24.78 (SD 15.57) and in the ≥ 70 years group was 9.89 (SD 11.41). Kruskal–Wallis test for OHIP-14 scores between the age groups was significant (*H*(3) = 9.64, *p* = 0.022). Post-hoc comparisons were not significant after application of the Bonferroni correction. When grouped for age, statistically significant differences were observed for the domains of functional limitation (*H*(3) = 8.94, *p* = 0.030), psychological discomfort (*H*(3) = 9.38, *p* = 0.025), physical disability (*H*(3) = 13.046, *p* = 0.0050), and psychological disability (*H*(3) = 11.15, *p* = 0.011). Mean scores for the different domains according to age group are presented in Figure 2. Neither DFS (*p* = 0.42) nor periodontal severity (*p* = 0.30) showed significant associations with OHIP-14 scores.

### Oral symptoms

In the FM group, 50% of participants reported oral mucosal symptoms, including ulcerations or blisters (30%), burning or stinging (21.7%), swelling (10%), and xerostomia (8.3%). Oversensitivity to food and drink resulting in oral problems was reported in 38.3% of participants, and 18.3% reported problems with toothpastes and mouthwashes. One participant used flavor-free toothpaste.

### Contact allergy

**Figure 3:**
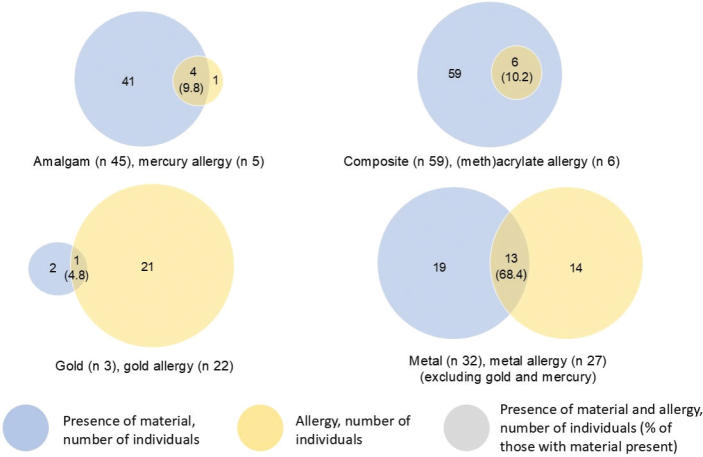
Illustration of the prevalence of contact allergy related to the presence of dental materials. *n*, number.

Prevalence of contact allergy to at least one contact allergen in the FM group was 73.3% (44 individuals). Allergies to substances in the Swedish baseline series were seen in 25 individuals (41.7%). Gold was the most prevalent contact allergy (23 individuals, 38.3%, followed by nickel (12 individuals, 20%). Table 3 shows frequencies of contact allergies for grouped allergens. Data for contact allergy related to dental material are shown in Figure 3. There was no correlation seen between burden of dental material and contact allergy. There was no relationship between reported oral symptoms and contact allergy in general or specific grouped allergens or observed mucosal lesions and contact allergy. There was no relationship between degree of periodontitis or dental caries and contact allergy.

**Table 3:** Prevalence of different contact allergies in the fibromyalgia participants, according to the allergen group.

Type of allergen	Number of allergies	Number of individuals (%)
Metals^a^	57	30 (50.0)
Fragrances^b^	27	17 (28.3)
(Meth)acrylates and associated substances^c^	10	6 (10.0)
Preservatives^d^	12	11 (18.3)

a Metals: Aluminium, cobalt, copper, gold, mercury, nickel, palladium, and tin.

b Fragrances: Carvone, cinnamal, eugenol, fragrance mix II, fragrance mix I, hydroperoxides of limonene, and hydroperoxides of linalool.

c (Meth)acrylates: Drometrizole, ethylene glycol dimethacrylate, hydroxyethyl methacrylate, methylhydroquinone, methyl methacrylate, tetrahydrofurfuryl methacrylate, and Tinuvin.

d Preservatives: Methyl dibromoglutaronitrile, sodium metabisulphate, and thimerosal.

## Discussion

This study aimed to assess oral health and oral health-related quality of life measures in FM. In this study, FM was significantly associated with severity of periodontal disease, with moderate periodontal disease being more common in the FM group than in the population controls. This difference was most apparent in younger individuals. Additionally, oral mucosal lesions were significantly more common in the FM individuals. OHIP-14 scores in the FM group were high, particularly in the younger participants. Regarding contact allergy, although the prevalence and burden of contact allergy was high, there was no apparent correlation between specific allergies and oral findings.

To date, very few comparative studies have been performed assessing oral health in FM, particularly with reference to periodontal disease. In this study, the significant association between FM and periodontal disease in the younger individuals (< 50 years) was an interesting observation, suggesting that younger individuals with FM may have additional risk factors in the development of an earlier periodontitis, with the difference becoming less pronounced with age. The reasons for this finding may include behavioral differences or inflammatory factors in the younger FM population. In this study, the FM group reported more use of dental floss and interdental brushes than the population controls. It may be the case that individuals with FM actually practice overly aggressive hygiene measures due to the high burden of oral symptoms and as an attempt to mitigate perceived oral problems, exacerbating gum recession. Whether there is a tendency to more aggressive oral hygiene practices in this group remains unanswered and should be explored further. Regardless, it is important that younger individuals with FM are examined and receive appropriate dental care.

A possible explanation for the observed association between FM and periodontal disease severity could be overlapping inflammatory pathways. It is increasingly described in the literature that peripheral factors can be contributory in FM, including inflammation. Low-grade systemic inflammation and altered pro-inflammatory cytokines have been observed in FM [2, 22–24]. Periodontal disease has been established to be a cause of low-grade systemic inflammation, with associations between periodontal disease and a multitude of systemic diseases with inflammatory etiology, including rheumatoid arthritis [25–29]. A bi-directional association between periodontitis and FM has been reported, supporting the hypothesis that systemic inflammatory mechanisms may underlie the relationship between the two conditions [30].

It is now widely accepted that the pain experienced in FM is due to ‘central sensitization’ – an exaggerated response of the central nervous system to nociceptive signals [2]. Many studies assessing oral health in FM focus on temporomandibular disorders (TMD) and oral discomfort as contributory factors in this mechanism [3, 4, 31, 32]. An association between periodontitis and reduced experimental peripheral pain tolerance has previously been described [33]. In this study, a number of other factors were identified that potentially could be sources of nociceptive and inflammatory input. The individuals with FM had a significantly higher prevalence of oral mucosal lesions. Lichenoid lesions were more frequent in the FM group, but this difference was not statistically significant (*p* = 0.057), which can be due to the small study sample. Associations between systemic inflammatory conditions and oral lichenoid lesions have previously been described, but there is no conclusive evidence of an association with FM [34, 35]. The lichenoid lesions in this study did not appear to be related to contact allergy to dental material, which is discussed further. Oral mucosal symptoms including xerostomia, discomfort, and burning sensations have previously been described in FM, but objective findings of oral pathology have been lacking [3]. Participants in this study reported oral discomfort, stinging, ulcerations, and xerostomia. Nervous system dysregulation in FM, as well as medications including anticholinergics and opiate analgesics, could potentially play a role in these symptoms. Objective measurement of xerostomia could be explored in further studies.

FM status was not associated with dental caries in this study. FM participants did not differ from controls in other measures such as number of teeth, education, and oral hygiene, with age being the predictor of cumulative caries burden. This suggests that the risk profile for the development of caries in the FM group does not differ from the general population. Xerostomia may also influence caries risk, but this was not reflected in the DFS measures in the present study. The aforementioned good oral hygiene practices in the FM group could also be contributory in the low caries burden in FM.

Assessment of oral health-related quality of life with use of the OHIP-14 questionnaire in the FM group demonstrated that FM individuals generally experience a poor quality of life. The overall OHIP-14 score of 14.27 compares unfavorably to previously reported levels in the Swedish general population of 4.30, as well as levels in individuals with severe periodontal disease (8.47) [18, 36]. Age was significantly related to OHIP-14 score, with younger individuals reporting higher OHIP-14 scores, while caries and degree of periodontal disease did not have an impact on scores. Sub-analysis of the different domains of the OHIP-14 showed that the age-related differences in scores were most pronounced in the domains related to functional and psychological disability rather than physical symptoms (pain). Interestingly, a retrospective analysis of previous questionnaire responses given by participants in this study regarding general pain and well-being showed that responses did not differ according to age (Hopkins et al., unpublished data) [8]. These combined findings would suggest that, in particular, younger individuals with FM may have multiple factors beyond oral disease, such as general health status and psychosocial factors, that play a role in perceived oral health impacts, and that oral symptoms impact specifically upon FM symptomatology in this group.

Regarding contact allergy in the FM participants, the prevalence of at least one contact allergy to the Swedish baseline series at 41.7% is considerably higher than reported in the general European population [37, 38]. Additionally, assessment of specific allergens showed high prevalence of allergy to dental materials including metals and acrylates. There was high concordance between acrylate allergy and oral exposure to acrylates, with all individuals with an acrylate allergy having composite fillings. Use of acrylic nails could, however, be a confounder here as rates of contact allergy to acrylates are usually low [39]. We have previously reported on the high levels of both acrylate and gold allergies in the FM population [7]. In this study, there was no association observed between oral symptoms and contact allergy or oral findings (periodontal disease, caries, oral lesions including lichenoid lesions) and contact allergy. An explanation for this can be the selection of dental material in the individuals examined in this study. Although the mean number of filled surfaces was representative for the age of the participants, there was a low prevalence of amalgam, gold and other metals, usually main culprits in allergy related to dental material [11, 40–43]. Additionally, 25% of participants answered that they previously had dental gold, but gold was only positively identified in 5% of participants, with many answering that gold had previously been removed either due to oral symptoms or loosening of material due to technical and/or biological complications with the prosthetic reconstruction. Previous or current dental gold was not associated with gold allergy in this study (*p* = 1.0). Our previous study found a significant association between gold allergy, dental gold, and oral symptoms [8] and a correlation between oral gold, gold allergy, and lichenoid lesions is proposed [44]. Additionally, avoidant practices regarding fragrance and taste substances could be employed in the FM group given that a high proportion of participants experienced hypersensitivity to foods and dental hygiene products. Interestingly, despite 18.3% in the FM group reporting sensitivity to oral products, only one individual used flavor-free toothpaste, and this individual had confirmed carvone allergy. We can conclude that FM individuals may have a tendency toward certain contact allergies related to oral exposure given the higher prevalence of oral lesions in the group and given the association between metal and fragrance allergy and oral lichenoid lesions [44, 45].

We acknowledge that there are several limitations that should be considered in the interpretation of the results of this study. The small sample size of FM individuals, and especially the skew toward older individuals, limits the power of the analyses. Of 119 individuals who participated in the original studies on contact allergy, 60 participated in the current study. Table 1 demonstrates that several key variables did not differ between the participants and nonparticipants, and one can therefore suggest that the current study was representative of the FM study group as a whole. However, it is possible that other unexamined factors such as willingness to undergo a dental examination due to perceived oral health difficulties or psychological factors influenced participation and therefore the results of the study. The control material was taken from an older study, and therefore caution should be exercised when comparing the groups. Due to the cross-sectional design of the study, it is difficult to ascertain a causal relationship between FM and periodontal disease. Lack of access to the participants’ medical and dental records meant that comprehensive information regarding dental interventions could not be identified.

This study has identified several potential areas where additional study is required. Longitudinal studies would enable clarification as to whether FM contributes to outcomes of periodontitis and vice versa. The use of the Hugoson and Jordan classification of periodontal disease was employed in the FM study group to allow comparison with the control material. Considering the findings concerning periodontitis and the hypothesized link with systemic inflammation, the use of current classification systems of periodontitis with a focus on the multifactorial and inflammatory nature of the condition should be considered in future study [46]. Likewise, the association between oral symptoms, including xerostomia, objective oral findings, and FM presentation, should be clarified. Contact allergy may also play a role in inflammatory status in both FM and periodontal disease, but studies on larger populations with relevant dental allergens are required to explore this link.

In conclusion, this study highlights an important association between FM and periodontal disease and oral mucosal health, which has previously been overlooked in the study of oral disease in FM. Dental practitioners should be aware that patients with FM may present at a younger age with periodontal disease, and particular attention should be paid to monitoring and prevention in this group. In addition, oral health and symptoms appear to have a direct impact on quality of life in FM patients. There is a need for further research into the underlying mechanisms linking FM and periodontal disease, including the role of systemic inflammation.

## Supplementary Material


