# Supplementary material for: Increased risk of periodontal disease and poor oral-related quality of life in fibromyalgia
Source: Acta Odontol Scand. 2026 Jul 31;85:46621. doi: 10.2340/aos.v85.46621 (PMC13440220; doi:10.2340/aos.v85.46621)
Supplement: Supplementary file 1 [file AOS-85-46621-s1.pdf]

Supplementary material has been published as submitted. It has not been copyedited or typeset by Acta Odontologica Scandinavica.

Supplements

Supplement 1: Patient questionnaire (translated from the original Swedish)

1. Are you sensitive to perfume? Yes ☐ No ☐
2. Have you had an itchy rash upon on exposure to perfume? Yes ☐ No ☐
3. Have you had airway difficulties on exposure to perfume? Yes ☐ No ☐
4. Are you sensitive to vehicle exhaust fumes? Yes ☐ No ☐
5. Are you sensitive to floral scents? Yes ☐ No ☐
6. Do you have or have you ever had pierced ears? Yes ☐ No ☐
7. Do you have or have you ever had other piercings? Yes ☐ No ☐
8. Have you ever had difficulties with itch, redness or swelling upon direct contact with gold objects such as earrings or rings? Yes ☐ No ☐
9. Have you ever had difficulties with itch, redness or swelling upon direct contact with non-precious metal jewellery or other metal objects? Yes ☐ No ☐
10. Do you use or have you used medication that contains gold? Yes ☐ No ☐
    a. If yes, when? ________________________________
11. Do you have or have you had gold in your mouth (fillings, crowns, bridges etc.)? Yes ☐ No ☐
    a. If yes, since when? ___________________________
    b. If it has been removed, when and why was this done?
    ____
12. Do you have or have you had any other type of filling, bridge, crown, implant or removable prosthesis? Yes ☐ No ☐
    a. If yes, which type and substance do you believe it to be?
    ____
    b. If it has been removed, when and why was this done?
    ____
13. Have you ever reacted with problems in the mouth after visits to the dentist? Yes ☐ No ☐
    a. If yes, describe
    ____
    b. Is there any substance or material that you suspect caused the problems?
    ____
14. Do you smoke? Yes ☐ No ☐
    a. If yes, how much __________________
15. Have you ever smoked? Yes ☐ No ☐
    a. If yes, when did you stop? ______________
16. Do you use ‘snus’? Yes ☐ No ☐
17. Have you ever used ‘snus’? Yes ☐ No ☐
    a. If yes, when did you stop? _______________

18. Do you use chewing gum regularly? Yes ☐ No ☐
19. Do you have any problems from the oral mucous membranes (inside of the mouth, cheeks)?
    Yes ☐ No ☐
    a. If yes, describe your problems
    ____
    b. Is there something that makes these problems better or worse? Please describe
    ____
20. Have you had problems with gum disease (periodontitis) or do you have any other condition or illness that affects the gums or mucous membranes? Yes ☐ No ☐
    a. If yes, describe your problems
    ____
21. Is there any food or drink that you do not tolerate due to oral symptoms? Tick all that apply
    a. Spices ☐
    b. Sweets ☐
    c. Chewing gum ☐
    d. Other, describe ______________________________
22. How often do you brush your teeth? Circle the answer that fits best

    Once a week   once a day   twice a day

23. Do you use the following:
    a. Toothpaste Yes ☐ No ☐
        i. Manufacturer/brand ____________________
    b. Electric toothbrush Yes ☐ No ☐
    c. Normal toothbrush Yes ☐ No ☐
    d. Floss/tape Yes ☐ No ☐
    e. Interdental brush Yes ☐ No ☐
    f. Toothpick Yes ☐ No ☐
    g. Fluoride mouthwash Yes ☐ No ☐
        i. Manufacturer/brand____________________
    h. Mouthwash Yes ☐ No ☐
        i. Manufacturer/brand____________________
24. Is there any dental product that you do not tolerate due to oral symptoms/problems?
    Yes ☐ No ☐
    a. If yes, describe
    ____

Supplement 2: The Oral Health Impact Profile (OHP-14).

Scores for each question: 0(never), 1 (hardly ever), 2 (occasionally) 3 (fairly often), 4 (very often). Maximum score 56.

Domain	Questions
1. Functional limitation	1. Have you had trouble *pronouncing any words* because of problems with your teeth, mouth or dentures?<br>2. Have you felt that your *sense of tast*e has worsened because of problems with your teeth, mouth or dentures?
2. Physical pain	1. Have you had *painful aching* in your mouth?<br>2. Have you found it *uncomfortable to eat any foods* because of problems with your teeth, mouth or dentures?
3. Psychological discomfort	1. Have you been *self-conscious* because of problems with your teeth, mouth or dentures?<br>2. Have you *felt tense* because of problems with your teeth, mouth or dentures?
4. Physical disability	1. Has your *diet been unsatisfactory* because of problems with your teeth, mouth or dentures?<br>2. Have you had to *interrupt meals* because of problems with your teeth, mouth or dentures?
5. Psychological disability	1. Have you found it *difficult to relax* because of problems with your teeth, mouth or dentures?<br>2. Have you been a bit *embarrassed* because of problems with your teeth, mouth or dentures?
6. Social disability	1. Have you been a bit *irritable with other people* because of problems with your teeth, mouth or dentures?<br>2. Have you had *difficulty doing your usual jobs* because of problems with your teeth, mouth or dentures?
7. Handicap	1. Have you felt that life in general was *less satisfying* because of problems with your teeth, mouth or dentures?<br>2. Have you been *totally unable to function* because of problems with your teeth, mouth or dentures?
